# An improved genome assembly of *Chrysanthemum nankingense* reveals expansion and functional diversification of terpene synthase gene family

**DOI:** 10.1186/s12864-024-10498-6

**Published:** 2024-06-12

**Authors:** Liping Jiang, Shi Chen, Xu Wang, Lin Sen, Gangqiang Dong, Chi Song, Yifei Liu

**Affiliations:** 1 Department of Pharmacy, Wuhan No.1 Hospital (Wuhan Hospital of Traditional and Western Medicine), Wuhan, 430022 People’s Republic of China; 2 College of Pharmacy, Hubei University of Chinese Medicine, Wuhan, 430065 People’s Republic of China; 3 Amway (China) Botanical R&D Center, Wuxi, 214115 P.R. China; 4 Institute of Herbgenomics, Chengdu University of Traditional Chinese Medicine, Chengdu, 610000 People’s Republic of China; 5 Hubei Provincial Key Laboratory of Chinese Medicine Resource and Chemistry, Hubei University of Chinese Medicine, Hubei, 430065 People’s Republic of China

**Keywords:** Genome, Chrysanthemum, Terpenes, TPS genes, Aromatic plant

## Abstract

**Background:**

Terpenes are important components of plant aromas, and terpene synthases (TPSs) are the key enzymes driving terpene diversification. In this study, we characterized the volatile terpenes in five different *Chrysanthemum nankingense* tissues. In addition, genome-wide identification and expression analysis of *TPS* genes was conducted utilizing an improved chromosome-scale genome assembly and tissue-specific transcriptomes. The biochemical functions of three representative TPSs were also investigated.

**Results:**

We identified tissue-specific volatile organic compound (VOC) and volatile terpene profiles. The improved *Chrysanthemum nankingense* genome assembly was high-quality, including a larger assembled size (3.26 Gb) and a better contig N50 length (3.18 Mb) compared to the old version. A total of 140 *CnTPS* genes were identified, with the majority representing the TPS-a and TPS-b subfamilies. The chromosomal distribution of these *TPS* genes was uneven, and 26 genes were included in biosynthetic gene clusters. Closely-related *Chrysanthemum* taxa were also found to contain diverse TPS genes, and the expression profiles of most *CnTPS*s were tissue-specific. The three investigated CnTPS enzymes exhibited versatile activities, suggesting multifunctionality.

**Conclusions:**

We systematically characterized the structure and diversity of *TPS* genes across the *Chrysanthemum nankingense* genome, as well as the potential biochemical functions of representative genes. Our results provide a basis for future studies of terpene biosynthesis in chrysanthemums, as well as for the breeding of improved chrysanthemum varieties.

**Supplementary Information:**

The online version contains supplementary material available at 10.1186/s12864-024-10498-6.

## Background

*Chrysanthemum* species are valued worldwide for their antioxidant, anti-inflammatory, and antimicrobial pharmacological properties [1]. The main chemical constituents thought to be responsible for these properties are terpenoids, phenolic acids, and flavonoids [2]. The perennial herb *Chrysanthemum nankingense*, a species closely related to *Chrysanthemum indicum*, is listed within the Pharmacopoeia of the People’s Republic of China as an anti-inflammatory treatment for hypertension and respiratory disorders. As a diploid plant, *Chrysanthemum nankingense* has been used as a model organism for genomic analysis and functional validation, and a draft genome has been released [3–6]. In South China, *Chrysanthemum nankingense* is also prized as a wild vegetable with a unique taste and aroma related primarily to its terpene content.

Terpenoids are largely responsible for the unique aroma of chrysanthemum flowers, foliage, and roots [1]. Terpene synthases (TPSs) are the key enzymes responsible for terpenoid biosynthesis, and are the primary drivers of terpene hydrocarbon skeleton diversity [7]. The *TPS* genes are categorized into two classes (I and II) based on their unique amino acid sequence motifs related to alternative catalytic strategies [8, 9]. Class I TPSs, which include several monoterpene and sesquiterpene cyclases, are characterized by a catalytically active α-helical domain (α domain) containing a pair of DDXXD and NSE/DTE motifs, as well as an unrelated, nonfunctional N-terminal α-helical domain (β domain) [7]. In contrast, class II TPS activity results from a DXDD motif located in the β domain, and which is necessary for the biosynthesis of phytohormones such as gibberellins [7, 10]. Alongside the β and α domains, the unified general TPS framework also includes an additional N-terminal γ domain, leading to a γβα tridomain architecture [7, 11]. Gene duplication and domain loss-related subfunctionalization and neofunctionalization are thought to be responsible for the functional diversity of plant TPSs [9].

Land plants contain seven different TPS subfamilies (TPS-a through TPS-h), with the TPS-e/f subfamily representing a merged clade [8, 12]. A recent phylogenetic analysis further divided these subfamilies into three groups corresponding to the TPS-c subfamily, the TPS-e/f subfamily, and the rest of the TPS-h/d/a/b/g subfamilies [9]. The TPS-c subfamily contains class II TPSs, including both bifunctional *ent*-copalyl diphosphate and *ent*-kaurene synthases (CPSKSs) and monofunctional *ent*-copalyl diphosphate synthases (CPSs) [8]. The TPS-e/f subfamily mainly includes class I *ent*-kaurene synthases (KSs) due to the loss of the DXDD motif in the β domain. The TPS-h/d/a/b/g subfamily is dedicated to secondary metabolism [9]. For example, the TPS-a subfamily contains sesqui-TPSs in both dicot and monocot plants, while the angiosperm-specific TPS-b subfamily contains monoterpene synthases. Members of both subfamilies contain the conserved N-terminal R(R)X_8_W motif. The TPS-g subfamily, while closely related to the TPS-b subfamily, contains acyclic mono-, sesqui-, and di-TPSs which lack the RRX_8_W motif [13]. The gymnosperm-specific TPS-d subfamily contains diverse mono-, sesqui-, and di-TPSs which can be further divided into the TPS-d1/d2/d3 subfamilies. The TPS-h subfamily is primarily found in lycophytes, mosses, liverworts, and ferns. Compared to the TPS-c and TPS-e/f subfamilies, the inclusion of bifunctional diterpene synthases in the TPS-h/d/a/b/g group (e.g., some members from the root TPS-h and TPS-d3 subfamilies) suggests that independent and parallel evolution occurred in this lineage from the common ancestral CPSKS gene copies of the three groups [9].

Our previous study of the *Chrysanthemum nankingense* genome provided the first survey of *TPS* gene diversity in chrysanthemums [6]. A substantial expansion of the *TPS* gene family in chrysanthemum may have contributed to terpenoid diversification, resulting in the diverse array of terpenoids found in extant species. Moreover, many *TPS* genes are grouped into biosynthetic gene clusters (BGCs) together with cytochrome P450-dependent monooxygenase (*CYP*) genes, suggesting that they are functionally related. With the recent publication of new *Chrysanthemum* genomes, including *Chrysanthemum lavandulifolium* [14], *Chrysanthemum seticuspe* [15], *Chrysanthemum makinoi* [16], and *Chrysanthemum morifolium* [17], it is now possible to conduct comparative analyses of *TPS* diversity across the genus. Such analyses will make functional validation of these diverse *TPS* genes possible.

Here, we present an improved, chromosome-scale *Chrysanthemum nankingense* genomic assembly. Our assembly was based on Oxford Nanopore technology (ONT) long reads and high-throughput chromatin conformation capture (Hi-C) paired-end reads. Furthermore, we combined metabolomics, transcriptomics, and functional genomics to comprehensively study the *Chrysanthemum nankingense* terpene synthases. The functions of the studied terpene synthases were verified in vitro and in vivo. Our results provide a solid basis for further studies of terpene biosynthesis in *Chrysanthemum nankingense*.

## Results

### Characterization of volatile terpenes in *Chrysanthemum nankingense*

**Fig. 1:**
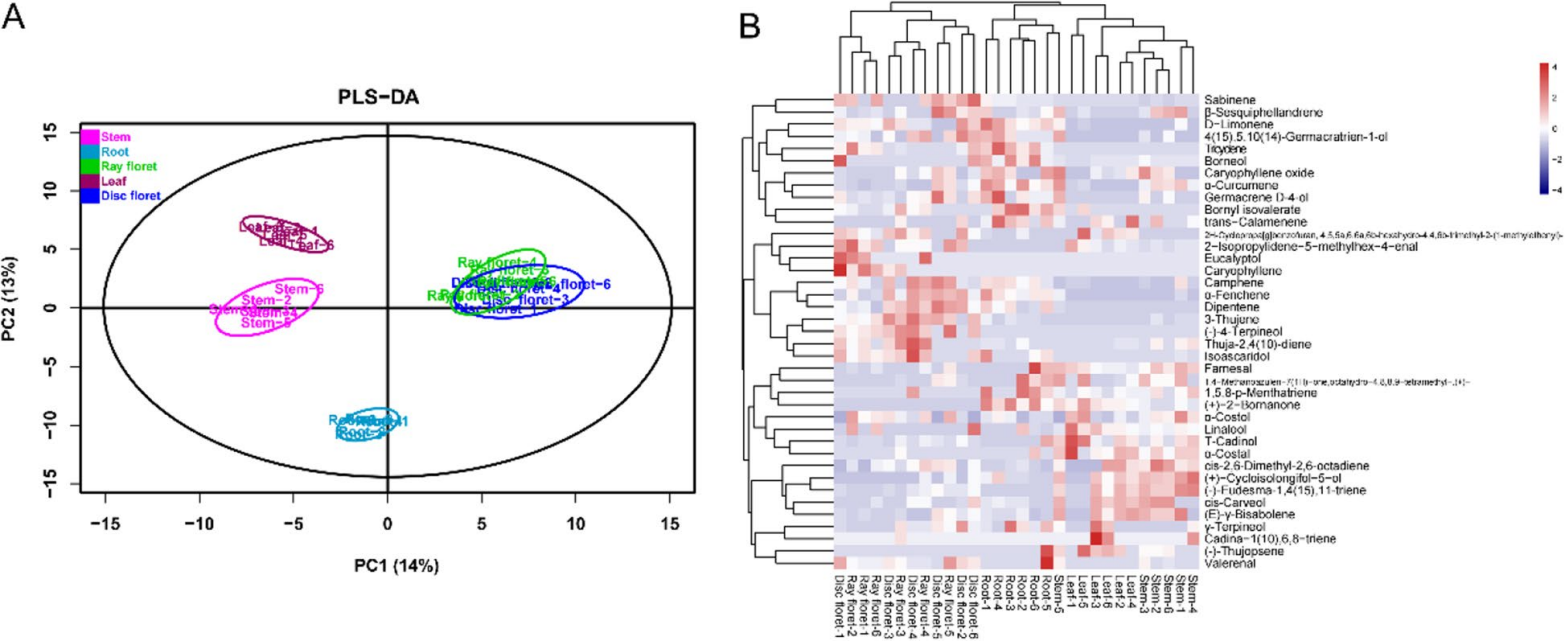
Volatile organic compound (VOC) profiles of five different *Chrysanthemum nankingense* tissues. **A** The first two principal components (PCs) of the PLS-DA analysis separated the tissue-specific VOC profiles. **B** Hierarchical clustering heat map of 39 volatile terpenes

The volatile organic compound (VOC) contents of five different *Chrysanthemum nankingense* tissues (disc floret, ray floret, leaf, stem, and root) were evaluated by gas chromatography-mass spectrometry (GC–MS) via headspace sampling. We identified diverse VOCs, including alcohols, terpenes, aldehydes, esters, hydrocarbons, acids, and benzenes, among others (Table S1). The partial least-squares discriminant analysis (PLS-DA) indicated that 27% of the total variation could be explained by the first two dimensions, PC1 and PC2 (Fig. 1A). The VOC profiles were found to be tissue-specific, with the exception of those from disc and ray florets, which could not be distinguished.

A total of 39 volatile terpenes were further characterized, including 19 monoterpenes (C10) and 20 sesquiterpenes (C15) (Table S2). Different tissues contained generally the same array of terpenoids, albeit at different concentrations. In particular, the contents of camphene, D-limonene, alpha-fenchene, borneol, beta-sesquiphellandrene, and alpha-curcumene were relatively high (> 1% relative abundance) (Fig. 1B, Table S2). Hierarchical cluster analysis indicated that different tissues contained significantly different terpene profiles, again with the exception of disc and ray florets. In general, flowers tended to contain relatively high concentrations of camphene, α-fenchene, dipentene, 3-thujene, (-)-4-terpineol, thuja-2,4(10)-diene, isoascaridol, and farnesal. In comparison, roots tended to contain relatively high concentrations of caryophyllene oxide, α-curcumene, germacrene D-4-ol, bornyl isovalerate, and trans-calamenene (Fig. 1B).

### Chromosome-scale *Chrysanthemum nankingense* genome assembly

**Fig. 2:**
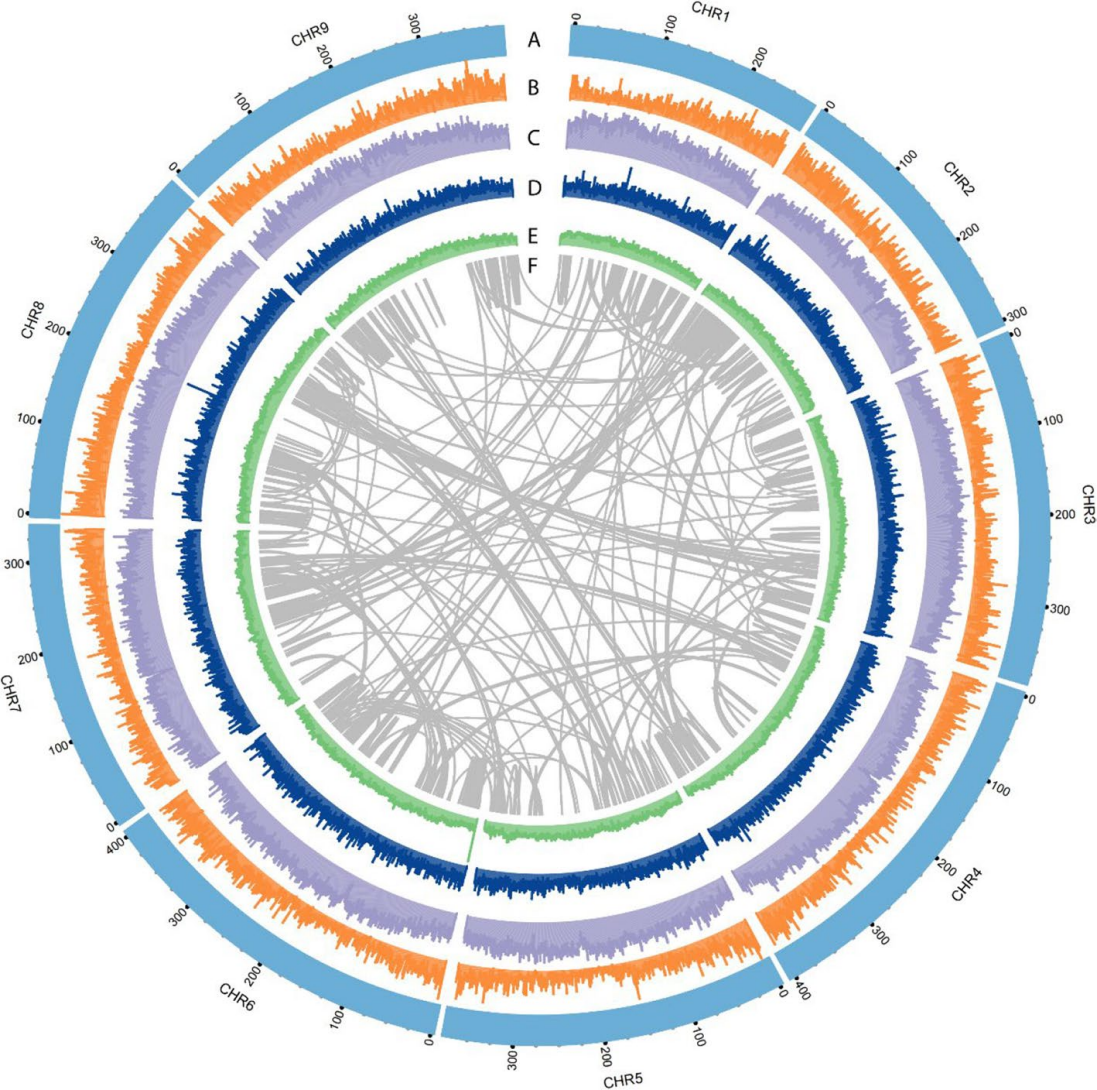
Chromosome-scale *Chrysanthemum nankingense* genome assembly. **A** Nine chromosome-scale pseudomolecules. **B** Gene density. **C** Density of DNA transposons. **D** Density of long retrotransposon terminal repeats. **E** GC content. **F** Intragenomic synteny analysis

The availability of high-quality reference genomes is crucial for the discovery and characterization of novel and useful plant genes. The previous *Chrysanthemum nankingense* draft genome (hereafter ‘version 1’) yielded 24,051 sequence contigs (N50 = 130.7 kb) with a total size of 2.53 Gb, representing 78–82% of an estimated genome size of 3.24 Gb (flow cytometry) or 3.07 Gb (19-mer analysis) [6]. In this study, we generated an additional 142.1 Gb of clean ONT reads, resulting in a total of 241.6 Gb (74.5 × coverage) of long reads at first assembly. After polishing using 477.5 Gb (147.4 × coverage) of clean Illumina short reads, we produced an assembly consisting of 3,708 contigs (N50 = 3.18 Mb) with a total size of 3.44 Gb. This assembly is slightly larger than the estimated *Chrysanthemum nankingense* genome size, suggesting genomic heterozygosity (from *k*-mers, 1.82%). Utilizing 1,208.4 million Hi-C paired-end reads, 3.26 Gb (94.8%) of the assembly was scaffolded into nine unambiguous pseudomolecules (chromosomes) and a chromosome-scale genome (version 2) was assembled, leaving 174 contigs unmapped (Fig. 2). The quality of the genomic assembly was found to be quite high, as evidenced by an Illumina short read mapping rate of 99.9% (Table S3) and a complete BUSCO gene recovery rate of 95.2% (Table S4). Overall, the quality of the assembly was comparable to that of other recently-released chromosome-level diploid chrysanthemum genomes (Table 1).

**Table 1:** Comparison of diploid *Chrysanthemum* genome assemblies

Statistics	*Chrysanthem-um nankingense* version 2	*Chrysan-themum nankingense* version 1	*Chrysa-nthemum lavandulifolium*	*Chrys-anthemum seticuspe*	*Chrysa-nthemum makinoi*
Total length (bp)	3,443,743,350	2,527,345,449	2,669,472,274	3042,490,000	3,113,668,257
Number of contigs	3,708	24,051	10,136	13,928	15,226
GC content	35.77%	35.62%	36.02%	35.5%	36.01%
N50 (bp)	3,182,889	130,678	496,998	1,027,000	258,200
N90 (bp)	411,372	52,399	136,070	-	-
Average (bp)	928,733.37	105,082.76	263,365.46	218,444.14	204,496.80
Median (bp)	433,008.5	72,931	-	-	-
Min (bp)	26,254	3,961	14,015	-	-
Max (bp)	34,662,800	1,664,587	4,500,000	-	-
Repeat content	74.57%	69.6%	66.15%	80.3%	80.4%
Predicted genes	74,172	56,870	64,257	620,134	95,064

The improved *Chrysanthemum nankingense* genome assembly enabled more robust repeat sequence identification: 74.6% in version 2 compared to 69.6% in version 1. The improved assembly contained 50.3% long terminal repeat retrotransposons (LTRs), 6.3% DNA elements, and 1.5% long interspersed nuclear elements (LINEs) (Table S5). We combined tissue-specific transcripts with ab initio gene prediction to annotate protein-coding genes across the genome. After quality control, a final set of 74,172 genes was retained (Table 1), which is considerably more than the 56,870 genes predicted in the version 1 genome [6]. However, no significant differences were observed for the average coding sequence length or the average number of exons per gene between the two assembly versions (Table S6). Approximately 96.4% of the genes were assigned to a predicted function, of which 89.9% had significant hits in the InterPro database (Table S7).

### Genome-wide identification of TPSs

**Fig. 3:**
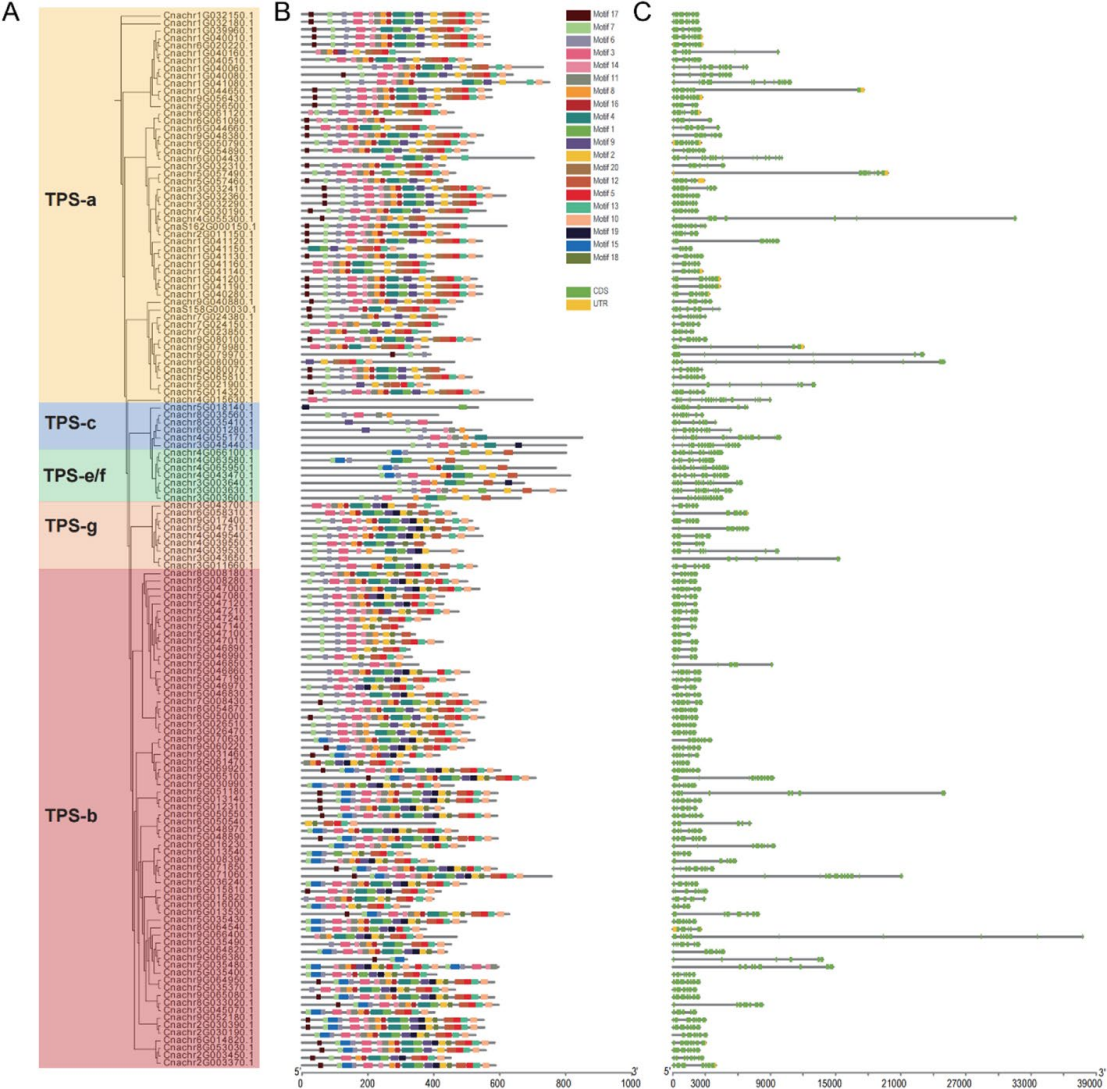
Characterization of *CnTPS* genes in *Chrysanthemum nankingense*. **A** Phylogenetic relationships among *CnTPS* genes. **B** Motif structures of *CnTPS* genes. **C** Exon–intron structures of *CnTPS* genes

Using the improved *Chrysanthemum nankingense* genome assembly, the *TPS* gene family was systematically investigated using a hidden Markov model (HMM) profile of the conserved C-terminal (PF03936) and N-terminal (PF01397) domains. A total of 140 *CnTPS* genes were obtained after removing redundant sequences (Fig. 3, Table S8). The *CnTPS* open reading frames (ORFs) varied from 921 (*Cnachr5G047141.1*) to 2553 bp *(Cnachr4G055170.1*) in length. The deduced amino acid (aa) length ranged from 306 (*Cnachr5G047140.1*) to 850 aa (*Cnachr4G055170.1*), with the corresponding molecular weight (Mw) ranging from 35.57 to 97.53 kDa. The theoretical isoelectric points (pIs) of the CnTPS proteins ranged from 4.85 (*Cnachr3G0036001*) to 9.93 (*Cnachr9G079971.1*). All CnTPS proteins were hydrophilic, with hydrophilicity values ranging from -0.717 (*Cnachr9G079970.1*) to -0.061 (*CnaS158G000030.1*). In addition, the aliphatic index (AI) of the CnTPS proteins varied from 70.54 (*Cnachr6G050540.1*) to 105.48 (*Cnachr8G008180.1*), and the instability index (II) varied from 26.9 (*Cnachr8G035560.1*) to 60.18 (*Cnachr1G044650.1*). A total of 82/140 CnTPS proteins were predicted to be localized to the chloroplast, 51/140 CnTPS proteins were targeted to cytoplasm, and 7 CnTPSs were extracellular (Table S8).

An unrooted neighbor-joining (NJ) tree was constructed to gain insight into the evolutionary relationships among *CnTPS* genes. All 140 identified CnTPS proteins were clustered into five previously-recognized TPS subfamilies: TPS-a (52), TPS-b (66), TPS-c (6), TPS-e/f (7), and TPS-g (9) (Fig. 3A). The majority of the *CnTPS* genes belonged to either the TPS-a or TPS-b subfamily, corresponding to a potential expansion of *CnTPS* genes encoding sesquiterpenes and monoterpenes, respectively.

MEME software was used to identify the conserved motifs of the CnTPS proteins. The top 20 conserved domains, including the R(R)X8W, DDxxD, NSE/DTE, and RxR motifs, were found to be especially common among TPS-a and TPS-b subfamily members (Fig. 3B). The arginine-tryptophan motif R(R)X8W (motif17), which plays a role in the isomerization cyclization reaction, was identified at the N-terminus of most *Chrysanthemum nankingense* TPS-a and TPS-b proteins. Two c-terminal aspartate-rich motifs, DDxxD (motif2) and NSE/DTE (motif14), are required to cleave the pentenyl diphosphate matrix by chelating Mg^2+^ or Mn^2+^. The DDxxD domain is the key terpene synthase functional domain, and serves as the stable Mg^2+^ binding site involved in the coordination of bivalent ions and water molecules. In *Chrysanthemum nankingense*, most TPS-a, TPS-b, TPS-e/f, and TPS-g subfamily members contained conserved DDxxD motifs, while TPS-c subfamily members lacked DDxxD motifs. Moreover, most CnTPS proteins were found to have at least one conserved DDxxD or NSE/DTE motif. The RxR motif (motif13) occurred only in the C-terminus of TPS-a, TPS-b, and TPS-g subfamily members, but was absent in TPS-e/f and TPS-c subfamily members.

The exon and intron structures of different TPS subfamily members were highly variable (Fig. 3C). For instance, the majority of TPS-a, TPS-b, and TPS-g subfamily genes contained from 5 to 8 exons and 4 to 7 introns, while the TPS-c subfamily genes contained from 15 to 16 exons and 14 to 15 introns. Gene structures among the TPS-e/f subfamily genes were also highly variable, containing between 7 and 14 exons. In general, CnTPS proteins within the same subfamily tended to possess similar motifs. The phylogenetic relationships among the CnTPSs are likely influenced by both motif location and gene structure.

### Chromosomal distribution of TPS genes and gene clusters

**Fig. 4:**
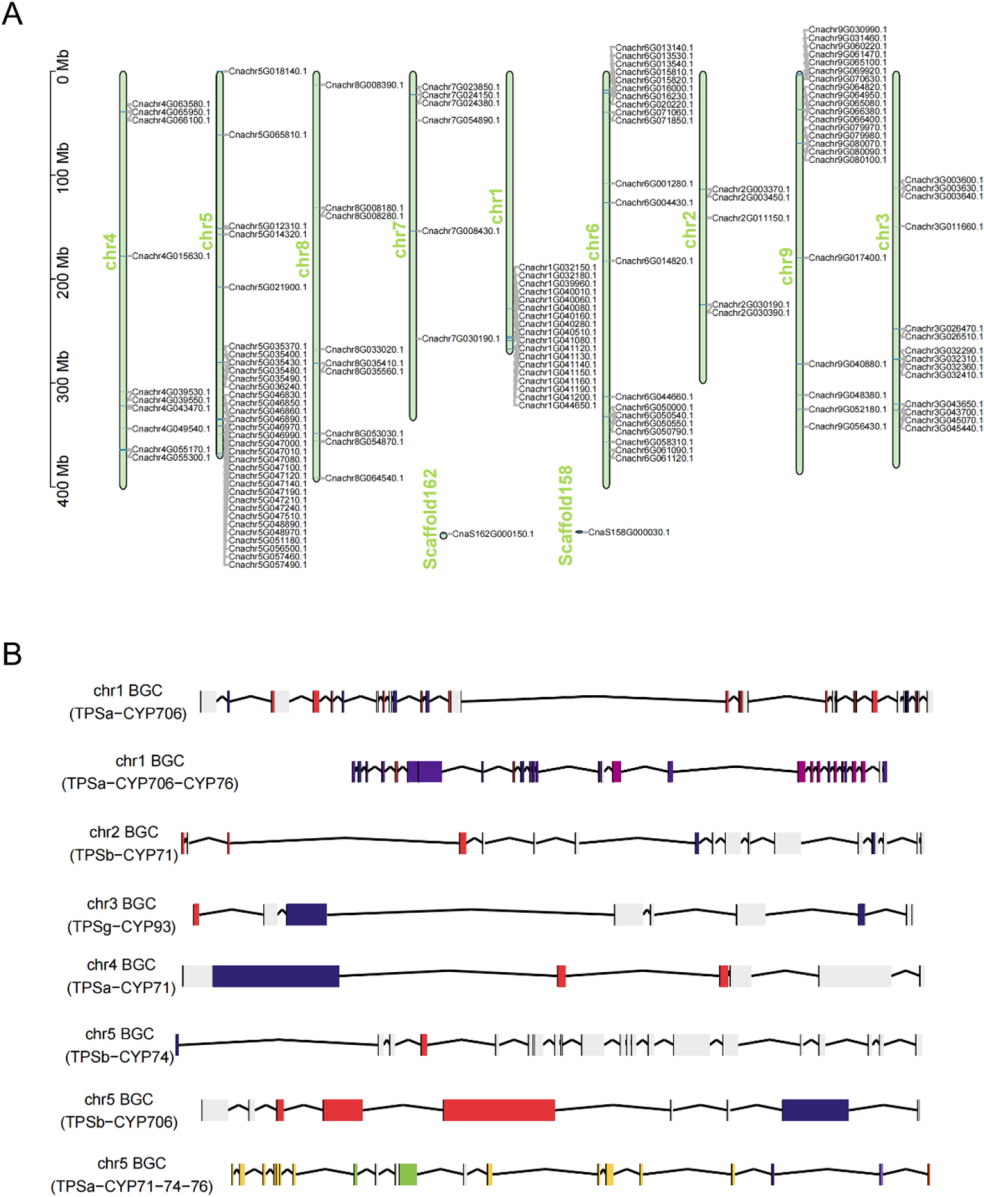
Chromosomal distribution of *CnTPS* genes and related biosynthetic gene clusters (BGCs). **A** Genomic locations of the identified *CnTPS*s. **B** Eight BGCs including both *CYP* and *TPS* genes

The majority of *CnTPS*s were unevenly distributed throughout nine chromosomes, with a maximum of 33 genes on chromosome 5 (chr5) (Fig. 4A). The two exceptions were *CnaS162G000150.1* and *CnaS158G000030.1*, which were positioned on two respective scaffolds. Many *CnTPS* genes were clustered, including 28 genes clustered on chr5, 18 on chr1, and 17 on chr9. These *CnTPS*s are widely believed to be associated with tandem duplication events occurring during the evolution of the *Chrysanthemum nankingense* genome.

Utilizing the chromosome-level genomic data, we further investigated *TPS* gene-related BGCs. A total of 64 BGCs were detected in the *Chrysanthemum nankingense* genome, in which 26 BGCs included the identified *CnTPS* genes and eight BGCs included both *TPS* and *CYP* genes (Table S9). Among the 26 BGCs including *TPS* genes, we identified eight and five BGCs on chr5 and chr6, respectively. Among the eight BGCs including both *TPS* and *CYP* genes, we identified three on chr5, two on chr1, and one each on chr2, chr3, and chr4 (Fig. 4B). These results imply that chr5 may be an important hotspot related to the evolution of TPS gene-related clusters in the *Chrysanthemum nankingense* genome.

### TPS gene diversity among closely-related *Chrysanthemum* species

Owing to its variable morphology, *Chrysanthemum nankingense* has been treated as either a variety of *Chrysanthemum indicum* or an independent species [18, 19]. Taxonomically, the *Chrysanthemum indicum* complex includes *Chrysanthemum indicum* var. *aromaticum* and the closely-related *Chrysanthemum lavandulifolium* [19]. To investigate the diversity and expression of *TPS* genes across taxa within the *Chrysanthemum indicum* complex, we conducted a comparative analysis using available genomic and transcriptomic data. The *Chrysanthemum nankingense* genome contained more *TPS* genes (140 *CnTPS*s), particularly TPS-b subfamily genes, than the *Chrysanthemum lavandulifolium* genome (108 *ClTPS*s) (Table S10). We further identified the expressed *TPS*s from multiple leaf-derived transcriptomes. The number of expressed *TPS*s varied greatly across taxa, with *Chrysanthemum lavandulifolium* exhibiting the fewest (22 *ClTPS*s) (Table S10). Interestingly, analysis of the expressed *TPS*s using multiple transcriptomes derived from different accessions revealed individual variation in both *Chrysanthemum indicum* and *Chrysanthemum indicum* var. *aromaticum* (Table S10). Such variable expression among individuals likely resulted from the interaction between genes and environments, leading to accession-specific expression patterns.

### Functional characterization of TPS genesin vitro and in vivo

**Fig. 5:**
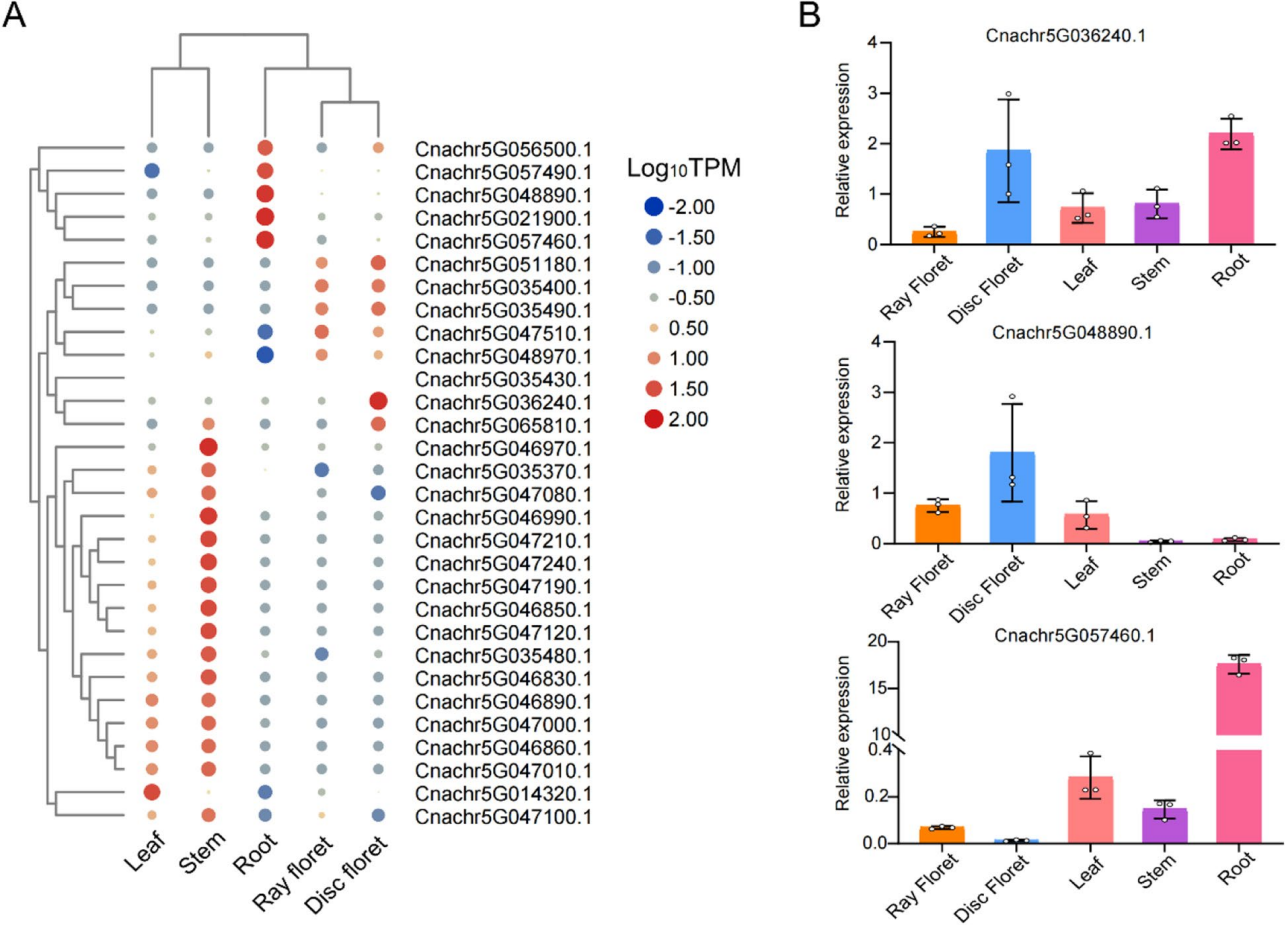
Expression profiles of *CnTPS* genes from chromosome 5. **A** Tissue-specific expression profiles of *CnTPS* genes from chromosome 5. **B** Quantitative real-time PCR (qRT-PCR) analysis of three representative genes

We used RNA sequencing (RNA-seq) to study the transcriptomes of five *Chrysanthemum nankingense* tissues (disc floret, ray floret, leaf, stem, and root). Overall, the *CnTPS* genes exhibited distinct tissue-specific expression profiles, suggesting their functional divergence in different tissues. For instance, of the 33 *CnTPS*s located on chr5, 30 genes exhibited differential expression between flowers (including both disc and ray florets), roots, and stems (together with leaves) (Fig. 5A). We therefore chose three *CnTPS* genes (*Cnachr5G036240.1*, *Cnachr5G048890.1*, and *Cnachr5G057460.1*) on chr5 for further functional investigation. Quantitative real-time PCR (qRT-PCR) analysis showed that *Cnachr5G036240.1* was ubiquitously expressed in roots, stems, leaves, and disc florets; while *Cnachr5G048890.1* was highly expressed in floral tissues, especially in disc florets; and *Cnachr5G057460.1* exhibited high expression exclusively in roots (Fig. 5B).

**Fig. 6:**
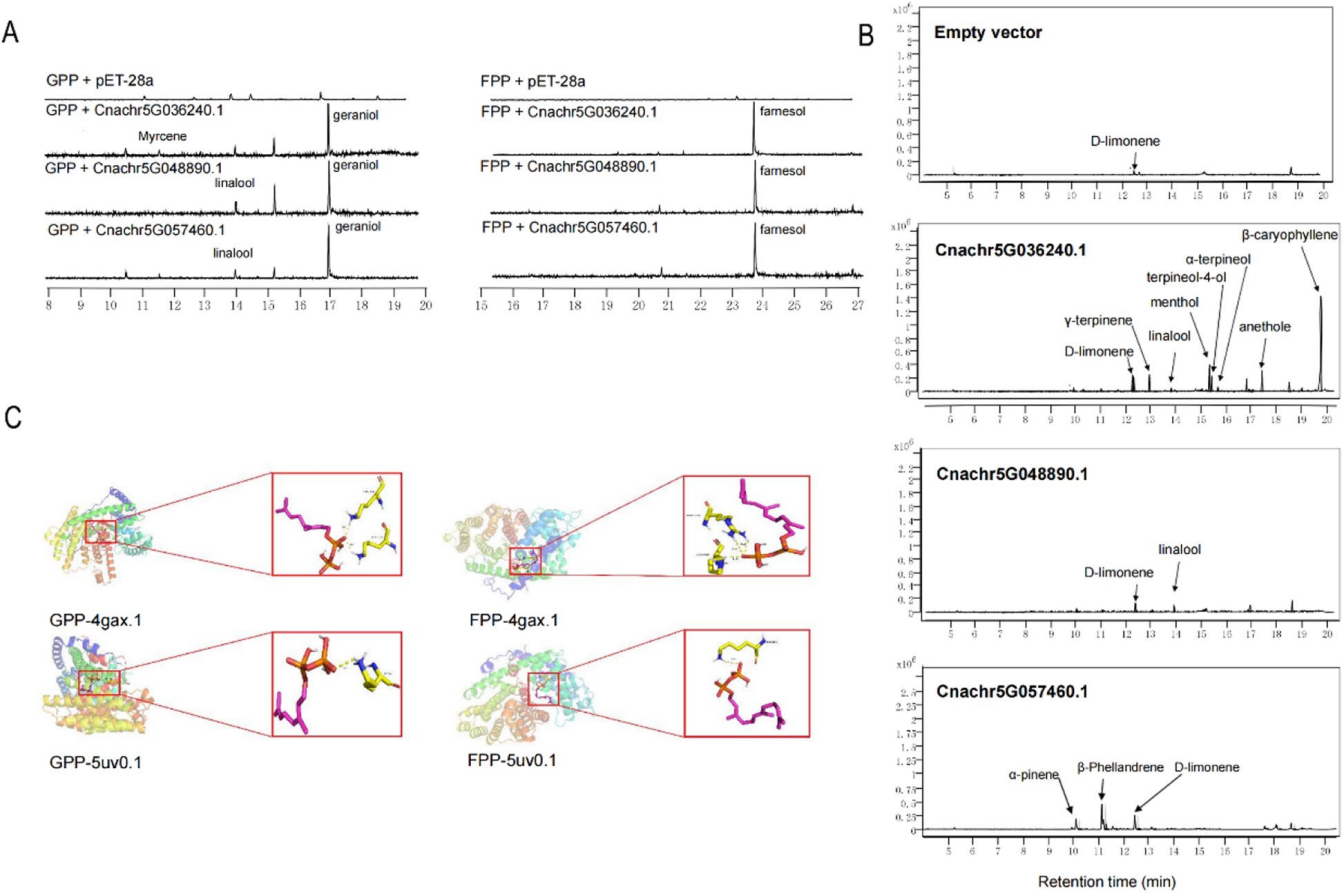
Functional characterization of three *Chrysanthemum nankingense* terpenoid synthases. **A** GC–MS analysis of the reaction products of GPP and FPP substrates, respectively. **B** GC–MS analysis of tobacco overexpression products. **C** The molecular docking of GPP and FPP with TPS protein

To validate the roles of these three *CnTPS*s in terpene biosynthesis, the enzymatic activities of three recombinant TPSs were evaluated in vitro. Each of the recombinant TPSs could synthesize monoterpenes (myrcene, linalool, geraniol) using geranyl diphosphate (GPP) as substrate and sesquiterpenes (farnesol) using farnesyl diphosphate (FPP) as substrate (Fig. 6A). In effect, a recombinant TPS protein can simultaneously catalyze GPP to produce two monoterpenes and catalyze FPP to produce farnesol. The in vivo transient overexpression of Cnachr5G036240.1 in tobacco leaves resulted primarily in the production of β-caryophyllene, anethole, menthol, γ-terpinene, and D-limonene, as well as small amounts of terpineol, linalool, and α-terpineol; the transient overexpression of Cnachr5G048890.1 in tobacco leaves resulted in the production of small amounts of limonene and linalool; and the transient overexpression of Cnachr5G057460.1 in tobacco leaves resulted in the production of β-phellandrene, α-pinene, and myrcene (Fig. 6B). Compared with the control, the types of terpenes expressed were unaltered, although their relative expression levels increased significantly (Fig. 6B). These results demonstrate that these three *CnTPS*s play a crucial role in terpene biosynthesis in *Chrysanthemum nankingense*.

### Molecular docking of CnTPSs with prenyl diphosphate substrates

To study the active sites of the investigated enzymes, the TPS model structure was evaluated with molecular docking using GPP (C10) and FPP (C15) substrates. The three TPSs were used to generate 2 protein models in Swiss Model, namely 4gax.1A and 5uv0.1.A (Table S11). Docking of the GPP and FPP substrates indicated that in each model protons were transferred to oxygen atoms on the substrate, resulting in hydrogen bond formation and reaction catalysis (except 1n24.1.A-GPP) (Fig. 6C). Specifically, GPP may be hydrogen-bonded with lysine to form a carbocation and FPP may interact with arginine or lysine to form a carbocation, prior to the formation of terpene products.

## Discussion

The fragrant *Chrysanthemum nankingense* contains monoterpene and sesquiterpene VOCs, including primarily camphene, D-limonene, caryophyllene, linalool, and their derivatives (Fig. 1). Terpenes are released from both vegetative and floral tissues to attract both pollinators and predators of attacking herbivores, and are directly toxic to herbivores and pathogens [20, 21]. Besides such ecological functions, these VOCs are responsible for the distinct scent associated with young *Chrysanthemum nankingense* leaves, making this species a popular vegetable in places such as Nanjing, China. Our study revealed tissue-specific VOC and terpene profiles, which will aid in the breeding of new varieties of highly-aromatic *Chrysanthemum nankingense.*

Next generation single-molecule sequencers with long-read capabilities have opened up a new era of genome assembly whereby the read lengths exceed those of most genomic repeats. Here, using an additional 142.1 Gb of clean ONT data, we assembled a chromosome-scale genome of *Chrysanthemum nankingense* (Fig. 2). The quality of this new assembly surpassed that of the older version 1 assembly [6] in terms of both assembled size (3.26 Gb vs. 2.53 Gb) and contig quality (N50 length: 3.18 Mb vs. 130 kb). In addition, the quality of our improved assembly is comparable to that of other recently-reported chromosome-level diploid chrysanthemum genomes, such as those for *Chrysanthemum lavandulifolium* [14] and *Chrysanthemum seticuspe* [15] (Table 1). As a potential progenitor of cultivated chrysanthemum, a chromosome-level genome of *Chrysanthemum nankingense* was also recently assembled to resolve the genomic origin and evolution of *Chrysanthemum morifolium* [17]. Both assemblies were similar, although different types of sequencing long-reads (ONT vs. PacBio SMRT) were used. Our new genomic data therefore provide an additional reference for future comparative genomic analyses.

Plants contain a medium-sized *TPS* gene family responsible for the biosynthesis of diverse terpene products, which constitute the largest class of plant secondary metabolites [9, 22]. Our new chromosome-scale assembly made possible the systematic identification of 140 *TPS* genes across the *Chrysanthemum nankingense* genome (Fig. 3). This is within the range of the number of *TPS* genes discovered in other plant genomes, e.g., 40 in Arabidopsis [23], 69 in grapevine (*Vitis vinifera*) [24], 44 in tomato [25], and 113 in *Eucalyptus grandis* [26]. Notably, *Chrysanthemum nankingense* contains more *TPS* genes than other Asteraceae species, including sunflower, lettuce, and artichoke [6]. We also identified variation in the number of *TPS* genes between different species and accessions within the *Chrysanthemum indicum* complex (Table S10). Given that chrysanthemums display ecological adaptations to a wide range of geographical regions, the generation of diverse terpenoids may be related to the independent evolution of different *TPS*s, resulting in species- or accession-specific *TPS* members. In a pan-genomic analysis of terpene synthases in rice, 13 variations of 45 *OsTPS* genes were identified, similarly suggesting diversification of *TPS* gene members between varieties within a species [27].

The distribution of *TPS* genes was uneven across the *Chrysanthemum nankingense* genome. Some hotspot regions were observed for homologous TPS clusters, such as those on chromosomes 5, 1, and 9 (Fig. 4). This may have resulted from gene replication events during the evolution of the *Chrysanthemum nankingense* genome [8]. Through bioinformatic prediction and BGC identification, we identified 26 BGCs which included *CnTPS* genes and eight BGCs which included both *TPS* and *CYP* genes (Fig. 4, Table S9). BGCs containing *TPS*s are prevalent in plants, especially *TPS/CYP* pairs [28]. Conserved *TPS/CYP* gene clusters may underlie terpene diversification in eudicots [29]. The *TPS/CYP* BGCs identified here therefore provide important data for future research on the origin and evolution of diverse terpenes in chrysanthemums.

In vitro and in vivo functional characterization of three *CnTPS*s (*Cnachr5G036240.1*, *Cnachr5G048890.1,* and *Cnachr5G057460.1*) demonstrated the versatilities of these CnTPSs in the biosynthesis of volatile terpenoids. Both Cnachr5G036240.1 and Cnachr5G048890.1 were predicted to be localized to the plastid, suggesting that they may be responsible for monoterpene biosynthesis. Cnachr5G036240.1 was able to produce the monoterpenes myrcene and geraniol, as well as the sesquiterpene farnesol, in vitro, and to catalyze the formation of seven monoterpenes and one sesquiterpene *in planta* (Fig. 6). Cnachr5G048890.1 produced the monoterpenes linalool and geraniol, and the sesquiterpene farnesol, in vitro, but only the monoterpenes linalool and limonene were obtained *in planta* (Fig. 6). Cnachr5G057460.1, a putative sesquiterpene synthase, was predicted to be localized to the cytoplasm. Cnachr5G057460.1 produced the monoterpenes linalool and geraniol, and the sesquiterpene farnesol, in vitro. However, this enzyme exhibited monoterpene synthase activity *in planta*, producing β-phellandrene, α-pinene, and D-limonene (Fig. 6). These findings are in line with our current understanding of the multifunctionality of plant TPSs, which can use one substrate (GPP or FPP) to produce diverse terpenes or use both substrates to generate monoterpenes and sesquiterpenes [30, 31]. The products of the in vitro assay may have differed from those produced *in planta* due to different biochemical conditions between plant and microbial expression systems [32, 33].

Among the three CnTPSs functionally characterized in this study, Cnachr5G036240.1 could produce the most diverse array of terpenes *in planta*, including seven monoterpenes (anethole, menthol, γ-terpinene, D-limonene, terpineol, linalool, and α-terpineol) and one sesquiterpene (β-caryophyllene)*.* Cnachr5G036240.1 was ubiquitously expressed in roots, stems, leaves, and disc florets (Fig. 5), suggesting that Cnachr5G036240.1 may play diverse roles in both pollination and defense. Cnachr5G048890.1, which was highly expressed in floral tissues (Fig. 5), exhibited unambiguous linalool synthase activity since linalool was produced both in vitro and *in planta.* These results suggest that Cnachr5G048890.1 may be responsible for the formation of floral scent and may play a pivotal role in attracting pollinators [30, 32, 34]. Cnachr5G057460.1, which was expressed exclusively in roots, produced β-phellandrene, α-pinene, and D-limonene *in planta*, suggesting a possible role in plant defense against insects and pathogens [35].

## Conclusions

In conclusion, we identified the volatile terpenes in five different *Chrysanthemum nankingense* tissues. Using our improved genome assembly, we also systematically identified *TPS* genes in the *Chrysanthemum nankingense* genome and those of closely related taxa. The *CnTPS* genes exhibited versatile biochemical functions, which may relate to their different roles in ecological adaptation. Our study provides an important reference for future research on terpene biosynthesis in chrysanthemums, as well as for breeding highly-aromatic plants.

## Materials and methods

### Sample collection

The formal identification of *Chrysanthemum nankingense* was carried out by Professor Hongbo Zhao from Zhejiang A & F University. Vouchers were stored in the exhibition center of the Chinese medicine specimens of Hubei University of Chinese Medicine (202006CNWH). In this paper, roots, stems, leaves, disc florets, and ray florets of *Chrysanthemum nankingense* were collected from the chrysanthemum germplasm resource nursery of Hubei University of Traditional Chinese Medicine, Wuhan, Hubei Province, China, in November 2020.

### Identification of VOCs by GC–MS

Six biological replicates (50 ± 1 mg) of five *Chrysanthemum nankingense* tissues (disc floret, ray floret, leaf, stem, and root) were collected in a headspace flask (20 mL). The VOCs were detected using a headspace solid-phase microextraction (SPME) system combined with GC–MS, using 2-octanol as the internal standard. In the SPME cycle of the PAL rail system, the incubation temperature was 60 °C, the preheating time was 15 min, the incubation time was 30 min, and the desorption time was 4 min. GC–MS analysis was performed on an Agilent 7890 gas chromatograph system coupled with a 5977B mass spectrometer. The system utilized a DB-WAX column injected in Splitless Mode, with helium as the carrier gas. The front inlet purge flow was 3 mL min^−1^ and the gas flow rate through the column was 1 mL min^−1^. The initial temperature was held at 40 °C for 4 min, then raised to 245 °C at a rate of 5 °C min^−1^, and held for 5 min. The injection, transfer line, ion source, and quad temperatures were 250, 250, 230, and 150 °C, respectively. The energy was -70 eV in electron impact mode. The mass spectrometry data were acquired in scan mode with an m/z range of 20–400 and a solvent delay of 0 min. Chroma TOF 4.3X software (LECO Corporation) and the Nist database were used to extract, align, and identify peaks; filter and calibrate data baselines; and deconvolute, integrate, and match spectra.

### Library construction and sequencing

The same *Chrysanthemum nankingense* plant used for our previous genomic sequencing [6] was also used in the present study. Total genomic DNA was isolated from fresh leaves using the CTAB method, with minor modifications. Total RNA was extracted from roots, stems, leaves, and flowers using a HiPure Plant RNA Kit (Magen, Guangzhou, China), according to the manufacturer’s instructions. A total of 10 μg of genomic DNA was used for Oxford Nanopore library preparation, using a previously-reported method [6].

The Hi-C libraries were prepared according to a previously-reported method [36]. Briefly, nuclear DNA was cross-linked in situ in 2% formaldehyde and then the nuclei were extracted and digested using the *Dpn*II restriction endonuclease. The sticky ends of the digested fragments were biotinylated, diluted, and ligated randomly. The biotinylated DNA fragments were enriched to construct the sequencing library. Sequencing was conducted on an Illumina NovaSeq 6000 platform to produce 150-bp paired-end reads.

### Genome assembly, quality assessment, and gene annotation

To construct a chromosome-scale *Chrysanthemum nankingense* genome assembly, we first used NextDenovo to assemble the Nanopore long reads. Next, we conducted two rounds of polishing, including using Racon (v1.4.11) [37] to polish the assembly with ONT reads and using Pilon (v1.23) [38] for iterative polishing with paired-end Illumina reads. Given the heterozygosity of many genomic regions, we further used Purge Haplotigs (v1.0.4) [39] to remove duplications, reassign allelic contigs, and improve the contig assembly. Finally, Hi-C reads which were uniquely mapped to the assembly were retained and corrected to generate contigs. These contigs were further linked into nine pseudo-chromosomes using the ALLHiC pipeline [40]. The Hi-C contact matrix was calculated and plotted with HiCExplorer (v2.1.1) [41].

Assembly quality was assessed by aligning the Illumina short reads to the genome using BWA (v0.7.17) [42]. Using the embryophta_odb10 and eukaryote_odb10 databases, Benchmarking Universal Single-Copy Orthologs (BUSCO, v4.1.4) was used to assess assembly quality and gene annotation according to the genome and protein modes, respectively. The structural and functional annotation of genes and the annotation of repetitive sequences were carried out according to a previously-reported strategy [6].

### RNA-seq and gene expression analysis

RNA-seq was performed on the Illumina nova-seq 6000 platform. After obtaining transcriptomic reads, Trimmomatic (v 0.39) [43] was used to remove the adapter sequences. Then, HISAT2 (v 2.0.5) [44] was used to map the trimmed sequences to the genome assemblies. StringTie (v 2.1.4) [45] was used to calculate Transcripts Per Million (TPM). Heat maps of tissue-specific gene expression profiles were created using TBtools (v 1.045) [46].

### Identification and characterization of *TPS* genes and BGCs

HMM search [47] and BLASTp [48] were combined to identity *TPS* gene family members using the conserved N-terminal (PF01397) and C-terminal (PF03936) domain sequences as queries against the *Chrysanthemum nankingense* genome. Results with E-values > 1e^−5^ were individually evaluated. In addition, gene homologs were obtained by running a local BLASTp search using previously-characterized proteins from Swiss-Prot as queries against all proteins with an E-value cut-off of 1e^−5^. Multiple sequence alignment was performed using Clustal Omega (v1.2.0) [49], with default parameters. The maximum likelihood tree was reconstructed using IQ-TREE [50]. Tree visualization and labelling were performed using the ggtree package in R [51]. Protein physicochemical properties were predicted using ExPASy [52]. Conserved motifs were analyzed using MEME SUITE [53]. Chromosome localization, gene structure, and heatmaps were visualized using TBtools. The plantiSMASH [54] tool was used to identify BGCs.

### Real-time qRT-PCR analysis

Collect five tissues during the peak flowering period of *Chrysanthemum nankingense*, including roots, stems, leaves, disc florets, and ray florets, for qRT-PCR analysis. Extract total RNA from various tissues of *Chrysanthemum nankingense* using the Polysaccharide Polyphenol Plant Total RNA Rapid Extraction Kit (BioTeke, RP3202). According to the manufacturer's instructions, reverse transcription was performed using PrimeScript™ RT Reagent Kit with gDNA Eraser (TaKaRa, RR047B) using an equal amount (1 μg) of total RNA. The mRNA copy data of *CnTPS* transcription in different tissues were corrected using the *Chrysanthemum nankingense* reference gene *Actin*. Design fluorescent quantitative primers for *CnTPS* gene using Primer 3 software [55]. Perform computational analysis using the 2^−ΔΔCt^ method and create charts using GraphPad Prism 8.3.0 for windows (GraphPad Software, San Diego, California USA, www.graphpad.com.).

### TPS expression and enzymatic activities in vitro

To evaluate in vitro enzymatic activity, *TPS* coding sequences were amplified from cDNA extracted from *Chrysanthemum nankingense* disc flower tissue. Each *TPS* gene was inserted into the *Eco*RI and *Hind*III sites of the prokaryotic pET-28a expression vector containing a His tag using a pEASY-Uni Seamless Cloning and Assembly Kit. The pET-28a:TPS construct was verified by complete gene sequencing, and was then transformed into the *Escherichia coli* strain ‘Rosetta’ (DE3).The recombinant protein was induced for 20 h using 0.1 mM IPTG at 16 °C. Next, the samples were ultrasonicated for 30 min and frozen at -80 °C. TPS enzyme activity assays were performed in 1.2 mL assay buffer (25 mM HEPES, pH7.5, 10 mM MgCl_2_, 10 μM MnCl_2_) containing 1 mL of ultrasonicated TPS protein and 12.5 μM of GPP/FPP. The reaction system was sealed with 0.3 mL n-hexane solution. The mixture was incubated at 30 °C for 1.5 h and then 45 °C for 15 min prior to collection of the synthesized volatiles. Negative control reactions were carried out with ultrasonicated protein from recombinant *E. coli* transformed with an empty pET-28a vector.

The monoterpene and sesquiterpene products in the n-hexane extract were evaluated by GC–MS. Then, the collected volatile gases were immediately transferred to the injection port (280 °C) of the GC–MS system (Agilent, 7890B-7000D) for 5 min of separation. Separation was performed under aseptic conditions on an HP-5MS Ultra Inert capillary column (30 mm × 0.25 mm × 0.25 mm), with helium as the carrier at a flow rate of 1.0 mL min^−1^. The temperature was held at 40 °C for 5 min, increased to 280 °C at a rate of 8 °C min^−1^, and then held for 5 min. Other settings included electron impact ionization (EI) at 70 eV, a 250 °C ion source temperature, and a 280 °C interface temperature. The mass spectrum was analyzed in the range of 40–400 atomic mass units. The National Institute of Standards and Technology mass spectral database (NIST14.L) was used to identify the mass spectra of the compounds. Standard samples were analyzed in the same way.

### Transient expression in *Nicotiana* tabacum

The *GUS* gene was excised from, and the *TPS* gene was inserted into, the pCAMBIA1301 vector to create the recombinant vector (PCAMBIA1301-TPS). The recombinant vector was transferred into *Agrobacterium tumefaciens* strain ‘GV3101’, which was then cultured on LB agar plates containing rifampicin (20 μg/mL) and kanamycin (50 μg/mL). The positive clones were incubated at 28 °C for two days, then inoculated into 30 mL of LB liquid medium containing appropriate hormones and cultured at 28 °C at 200 rmp to OD_600_ = 0.8–1.0. *Agrobacterium* was suspended in injection buffer (10 mM MES, 10 mM MgCl_2_, 0.2 mM acetosyringone) until OD_600_ = 0.8–0.9, incubated at room temperature for 2–4 h, and injected into the back of the youngest three leaves of 25-day-old tobacco. The transformed plants were kept in a growth chamber at 28 °C and exposed to long day conditions (16/8 h light/dark). Leaves were collected 5–7 days after transformation. Exactly 1.5 g of crushed tobacco leaves were collected and mixed with 2 g of NaCl and 2.0 mL of water. Next, 10 mL of n-hexane was added and the mixture was vortexed and subjected to ultrasonic extraction for 30 min. After removing the water from the n-hexane layer with anhydrous sodium sulfate, nitrogen was blown to 0.2 mL for GC–MS detection.

### Supplementary Information

Supplementary Material 1.

## Data Availability

The authors declare that the data supporting the findings of this study are available within the article and its supplementary information files. The raw sequences and genome have been deposited in the National Genomics Data Center (NGDC), China National Center for Bioinformation (CNCB) database with the BioProject accession PRJCA008358.

## Abbreviations

TPS: Terpene synthase
VOC: Volatile organic compound
CPSKS: *Ent*-Copalyl diphosphate and *ent*-kaurene synthase
CPS: *Ent*-Copalyl diphosphate synthase
KS: *Ent*-Kaurene synthase
BGC: Biosynthetic gene cluster
CYP: Cytochrome P450-dependent monooxygenase
ONT: Oxford Nanopore technology
Hi-C: High-throughput chromatin conformation capture
PLS-DA: Partial least-squares discriminant analysis
LTR: Long terminal repeat retrotransposon
LINE: Long interspersed nuclear element
HMM: Hidden Markov model
ORF: Open reading frame
Mw: Molecular weight
pI: Isoelectric point
AI: Aliphatic index
NJ: Neighbor-joining
GC–MS: Gas chromatography-mass spectrometry
TPM: Transcripts Per Million
